# Designing health messages for older adults: factors that influence older adults’ perceptions of hope for making health behavior changes

**DOI:** 10.15460/jlar.2024.2.2.1377

**Published:** 2024-10-30

**Authors:** Amber K. Worthington, Britteny M. Howell, Allexis Mahanna, Najma A. Musa

**Affiliations:** University of Alaska Anchorage, United States

**Keywords:** hopeful language, hope-based messaging, persuasive hope theory, strategic message design, health promotion

## Abstract

One motivator for improving older adults diet and exercise may be perceptions of hope; however, little is known about what factors influence hope in older adults and what linguistic features can subsequently be used in strategic messages to increase older adults feelings of hope. Using Persuasive Hope Theory (PHT), this study examined whether older adults self-reported self-efficacy and perceptions that improving their diet and exercise are important, possible, congruent with their goals (i. e., goal congruence), and will lead to a better future (i. e., future expectation) influence self-reported feelings of hope. A convenience sample of older adults (N = 58) in Southcentral Alaska completed a questionnaire. A multiple regression analysis revealed that importance and future expectation significantly and positively predicted feeling hopeful about fruit and vegetable in-take; possibility, goal congruence, and self-efficacy did not. The PHT variables for increasing exercise did not significantly predict feeling hopeful about increasing exercise. Participants reasons for feeling hopeful included excitement and motivation about a supportive environment; some participants reported not feeling hopeful due to perceived frailty or illness. These results are an important first step towards understanding what factors and subsequent hope-based language choices motivate older adults to engage in positive health behaviors via feelings of hope.

## 1 Introduction

The proportion of older adults in the United States (US) is increasing. In fact, by 2034, the number of adults aged 65 and older (i. e., 77.0 million) is projected to exceed the number of children under age 18 (i. e., 76.5 million) for the first time in history (U.S. Census Bureau 2018). These demographic trends are evident in Alaska, which has the highest proportion of older adults of any state in the US (Administration on Aging 2018), as well as some of the greatest geriatric provider shortages per capita in the country (United Health Foundation 2020; Kaiser Family Foundation 2022). Further, the proportion of Alaska Native/American Indian peoples aged 65 years and older is expected to increase from 10% in 2010 to 16.8% by 2050 (Vincent and Velkoff 2010). Alaska Native/American Indian peoples have shown remarkable resilience in the face of forced assimilation and cultural suppression (Burnette and Figley 2017); however, persistent disparities in health underscore the need to promote health and healing for Alaska Native/American Indian peoples and communities (Crouch et al. 2023). Thus, it is imperative to study ways to improve successful aging across the US (Pruchno 2015; Bernhold 2021) and specifically within Alaska (Howell et al. 2022; Worthington et al., forthcoming).

Successful aging is facilitated by engaging in health-related behaviors, including consuming a high-quality diet and engaging in recommended levels of physical activity (Gopinath et al. 2016; Gopinath et al. 2018); however, previous research has found that older adults in the US may struggle to maintain high dietary quality (Nicklett and Kadell 2013) and recommended levels of physical activity (Sparling et al. 2015; Watson et al. 2016). This is also the case in Alaska, where older adults experience barriers to obtaining healthy dietary intake and physical activity (Cobb, Espey, and King 2014; Garrett et al. 2015; Mayeda et al. 2016).

Lifespan perspectives indicate that appropriate language choices to motivate behavior change must recognize the important role that aging plays in how language use and preferences across the lifespan change and adapt (Nussbaum and Worthington 2017). Older adults favor positive language in health messages (e.g., Liu et al. 2019), which suggests that messages using linguistic content that leads to positive feelings may be particularly beneficial as a motivational tool for older adults (see Nussbaum and Worthington 2017). One key motivator for improving older adults diets and physical activity levels may therefore be perceptions of hope (Nussbaum and Worthington 2017; Worthington et al., forthcoming). Indeed, Persuasive Hope Theory (PHT) (Chadwick 2015) posits that hope is a discrete, future-oriented emotion that motivates behavior by focusing peoples thoughts on opportunities for action that will lead to future rewards. A recent review with 36 studies found that psychological interventions (e.g., cognitive behavioral therapy) can increase older adults feelings of hope (Hernandez and Overholser 2021); however, incorporating PHT into health messages for older adults may present a more cost-effective way to increase older adults feelings of hope more widely. Thus, it is important to determine if health messages with hope-based linguistic features can increase older adults feelings of hope and thereby positively influence older adults health behaviors; however, a critical first step is to determine which hope-based factors lead to feelings of hope in older adults. This study therefore examined whether perceptions of importance, goal congruence, possibility, future expectation, and self-efficacy from PHT influence older adults feelings of hope. The results have important implications for what positive, hope-based linguistic features (Worthington et al., forthcoming) can be used to effectively design health messages that increase older adults feelings of hope and subsequently increase their motivation to make positive health behavior changes.

## 2 Background

Factors that motivate behavior change shift over the lifespan (Nussbaum and Worthington 2017; Notthoff and Carstensen 2014). Substantial work grounded in Socioemotional Selectivity Theory (SST) has demonstrated that motivation changes across adulthood by shifting from goals related to preparation, exploration, and information seeking in young adulthood to goals related to emotional satisfaction in older adulthood (Carstensen, Isaacowitz, and Charles 1999). SST posits that time horizons are the key mechanism underlying these changes (Fredrickson and Carstensen 1990; Carstensen, Isaacowitz, and Charles 1999). In young adulthood, time horizons are perceived as open-ended, and people therefore prioritize preparation, exploration, and information seeking. As people get older and time horizons grow shorter, they increasingly prioritize emotional satisfaction (Lang and Carstensen 2002; Fung and Carstensen 2006).

Older adults prioritization for emotional satisfaction is linked to an age-related positivity effect in cognitive processing (Reed, Chan, and Mikels 2014; Reed and Carstensen 2012; Notthoff and Carstensen 2014). Indeed, older adults prefer, attend to, and remember positive language and information better than negative compared to younger people (Reed and Carstensen 2012). This also appears to be the case with the language in health information, as Shamaskin, Mikels, and Reed (2010) found that older adults prefer and better remember health brochures with positive language about the benefits of healthy lifestyles over those with negative language warning about the risks of unhealthy lifestyles. Likewise, Notthoff et al. (2016) found that older adults rated positive language about physical activity as more motivating than negative language. Notthoff and Carstensen (2014) further found that positive language also significantly increased older adults actual health behaviors (i. e., walking) and behavioral maintenance compared to negative language.

Positive language and information may thus be particularly motivating for older adults via positive affect. Indeed, Liu et al. (2019) found that gain-framed physical activity messages lead to positive affect, and older adults found messages that made them feel positive to be more effective (whereas younger adults found messages that made them feel negative to be more effective). This idea also relates to Persuasive Hope Theory (PHT) (Chadwick 2010, Chadwick 2015).

### 2.1 Persuasive Hope Theory

PHT (Chadwick 2010, Chadwick 2015) defines hope as a discrete, future-oriented emotion that motivates behaviors by directing thoughts towards opportunities for action that enable the achievement of future rewards. This motivational component of hope may help explain the persuasiveness of positive language for older adults (Nussbaum and Worthington 2017). PHT posits that for a message to elicit feelings of hope, it must evoke perceptions of the opportunity for action and the efficacy of a specific recommended behavior to achieve a desired future outcome. Appraisals of opportunity are evoked by perceptions of importance, goal congruence, possibility, and future expectation (Chadwick 2015).

Perceptions of importance include an assessment of whether the desired future outcome is personally relevant. Perceptions of goal congruence include an assessment of whether the desired future outcome is consistent with someones personal goals or motives. Perceptions of possibility include an assessment of whether the desired future outcome is likely to transpire. Perceptions of future expectation include an assessment of whether the desired future outcome would result in a better future. Perceptions that there is a low opportunity for the desired future outcome (i. e., low appraisals of importance, goal congruence, possibility, and / or future expectation), will not lead to feelings of hope. On the other hand, perceptions that there is a high opportunity for the desired future outcome (i. e., high appraisals of importance, goal congruence, possibility, and future expectation), will lead to feelings of hope (Chadwick 2015). This study therefore poses the following hypotheses:

- H1: Older adults perceptions of (a) importance, (b) goal congruence, (c) possibility, and (d) future expectation for increasing fruit and vegetable intake are positively
- H2: Older adults perceptions of (a) importance, (b) goal congruence, (c) possibility, and (d) future expectation for increasing physical activity are positively related to feelings of hope about physical activity improvements.

According to PHT, appraisals of efficacy include perceptions of self-efficacy (Chadwick 2015). Perceptions of self-efficacy include an assessment of whether someone believes they can perform the recommended behavior. For example, an older adult with high nutrition self-efficacy would believe that they are capable of shopping for, preparing, and consuming sufficient fruits and vegetables; an older adult with low nutrition self-efficacy would not. PHT posits that low self-efficacy will not contribute to feelings of hope, whereas high self-efficacy will contribute to feelings of hope. Self-efficacy may be particularly important for older adults to feel hopeful, as substantial research has indicated that older adults have low self-efficacy to engage in health-related behaviors (Bennett and Gaines 2010; Nilsson, Lundgren, and Liliequist 2012; Bardach, Schoenberg, and Howell 2016). This study therefore poses the following hypotheses:

- H3: Older adults perceptions of self-efficacy for increasing fruit and vegetable intake is positively related to feelings of hope about these dietary improvements.
- H4: Older adults perceptions of self-efficacy is positively related to feelings of hope about physical activity improvements.

The use of PHT for older adult lifestyle behavior change is an emerging area of research; thus, this study further used Grounded Theory (Charmaz 2006) to qualitatively investigate the reasons older adults gave in response to whether they felt hopeful about making changes to their fruit and vegetable intake or their physical activity patterns. This study therefore considered:

RQ1: What are the reasons older adults give for their perceptions of hope related to increasing fruit and vegetable intake and getting more physical activity?

## 3 Materials and methods

### 3.1 Participants and procedures

Participants (N = 58) were recruited through four older adult independent-living housing communities in urban Southcentral Alaska as part of an intervention study to improve diet and exercise patterns (Worthington et al., forthcoming), which were community-identified needs (Howell et al. 2022). Participants completed a baseline questionnaire that assessed perceptions of importance, goal congruence, possibility, future expectation, self-efficacy, and hope related to both increasing fruit and vegetable intake and getting more physical activity. The questionnaire also included demographic questions and an open-ended question regarding perceptions of hope. All study protocols and instruments were reviewed and approved by Southcentral Foundation and the Alaska Area Institutional Review Board (AAIRB project #2021-08-038). This manuscript was also reviewed and approved by Southcentral Foundation, the Alaska Native-owned health corporation serving nearly 70,000 Alaska Native and American Indian peoples living in Anchorage, the Matanuska-Susitna Borough, and 55 rural villages in the Anchorage Service Unit.

- PHT measures. Perceptions of importance were assessed with the items “Increasing the amount of fruits and vegetables I eat is **important** to me” and “Getting more physical activity is **important** to me.” Perceptions of goal congruence were assessed with the items “Increasing the amount of fruits and vegetables I eat is **a goal** for me” and “Getting more physical activity is **a goal** for me.” Perceptions of possibility were assessed with the items “Increasing the amount of fruits and vegetables I eat is **possible** for me” and “Getting more physical activity is **possible** for me.” Perceptions of future expectation were assessed with the items “Increasing the amount of fruits and vegetables I eat will **make my future better**” and “Getting more physical activity will **make my future better**.” Feelings of hope were assessed with the items “Right now, I feel **hopeful** about increasing the amount of fruits and vegetables I eat” and “Right now, I feel **hopeful** about getting more physical activity.” Response options for all items ranged from 1 = Strongly disagree to 5 = Strongly agree. In a recent paper, Matthews, Pineault, and Hong (2022) conclude that single-item measures often show good psychometric properties (e.g., good reliability and criterion-related validity) and are preferred over multiple-item measures when survey fatigue is likely. Older adults are at high risk for survey fatigue (Le, Han, and Palamar 2021); thus, single-item measures were used in this study.
- Self-efficacy measures. Self-efficacy was measured with items based on the Self Rated Abilities for Health Practices Scale (SRAHP: Becker et al. 1993). Participants were asked to respond to eight total items designed to specifically assess nutrition and exercise self-efficacy, with response options ranging from 0 = “not at all” to 4 = “completely”. Nutrition self-efficacy was assessed with the following items:
  1. I am able to find healthy foods that are within my budget;
  2. I am able to figure out how much I should weigh to be healthy;
  3. I am able to tell which foods are high in fiber content;
  4. I am able to drink as much water as I need to drink every day (range = 0–4; = .80).

Exercise self-efficacy was assessed with the following items:

1. I am able to do exercises that are good for me;
2. I am able to find ways to exercise that I enjoy;
3. I am able to know when to quit exercising;
4. I am able to keep from getting hurt when I exercise (range = 0–4; = .84).

Of note, we did not use the actual SRAHP; however, this study selected a subset of relevant items from the SRAHP with which we created our own measures for nutrition and exercise self-efficacy because older adults are at high risk for survey fatigue (Le, Han, and Palamar 2021).

- Open-ended hope question. After answering the questions “Right now, I feel **hopeful** about increasing the amount of fruits and vegetables I eat” and “Right now, I feel **hopeful** about getting more physical activity,” participants were asked “Please explain why or why not” with an open text entry option.

#### 3.1.1 Data analysis

The quantitative data for the hypotheses were analyzed using descriptive statistics, bivariate correlations, and multiple linear regressions in SPSS version 27.0 (IBM Corporation 2020). A power analysis was conducted using G*Power version 3.1.9.7 (Faul et al. 2007) that indicated the required sample size to achieve 80% power at a significance criterion of *α* = 0.05, is N = 49 for a multiple linear regression with five predictors. Thus, the obtained sample size of N = 58 is adequate.

Written comments to the question about feeling hopeful to make changes to fruit and vegetable intake or exercise were coded using line-by-line textual analysis in NVivo 12 Pro (QSR International 2018) using the principles of Grounded Theory (Charmaz 2006). This framework allows for the systematic analysis of data with open coding and an inductive approach to identify common concepts (Glaser and Strauss 1967). Open coding (i. e. initial coding) entails sorting and labeling data and allows for the systematic comparison of qualitative responses and is considered the basis of Grounded Theory (Charmaz 2006). Using inductive analysis with a grounded theory framework, low-inference codes were attached to text segments that came organically from the data (Card 2015). Low-inference codes are those that are discrete from each other and do not require high levels of value judgments to assign text segments to codes. For example, a code for “transportation problems” and a code for “health status” require a low-level of inference because they are distinct categories that would not often be confused. The data was organized into common themes for constant comparison that are driven by the data rather than attempting to fit the data into existing theoretical frameworks.

The second author and study PI (an anthropologist) coded the first 10 responses to the question, creating and refining a first draft of the codebook. The fourth author (a trained health sciences student) then coded all written responses (n = 43, since not all participants responded to the open-ended question) using the draft codebook, provided feedback, and discussed changes with the PI and edits that refined the second version of the codebook. The PI then coded the remaining 33 responses and updated the codebook a third time. Lastly, the 3rd and final draft of the codebook was discussed with the student co-coder (i. e., the fourth author), who then used it to verify all comments in the dataset were coded and discuss any remaining discrepancies. The PI then calculated inter-rater agreement; the coding team achieved 86% inter-rater agreement, which is considered reliable for qualitative data analysis (Neuendorf 2002; Bernard 2006; Morse 2015).

## 4 Results

A total of 58 older adults completed the survey. Participants ranged in age from 57 to 87 (M = 71.98, SD = 8.74). Participants self-identified as female (n = 49, 84.48%) and male (n = 9, 15.52%). Participants self-identified as White / Caucasian (n = 39, 67.24%), Alaska Native / American Indian peoples (n = 14, 24.14%), Asian (n = 1, 1.72%), Black / African American (n = 8, 13.79%), and Native Hawaiian / Other Pacific Islander (n = 1, 1.72%). We appreciate all our participants and recognize that Alaska Native / American Indian peoples may distrust research for multifaceted reasons including their history of forced assimilation, discrimination, and other mistreatment (Buchwald et al. 2006). Therefore, we further appreciate the willingness of the participants who identify as Alaska Native / American Indian peoples to complete the study. Additional participant demographics are displayed in Table 1.

### 4.1 Hypotheses 1 and 3: Hope for increasing fruit and vegetable intake

Hypothesis one predicted that older adults perceptions of (a) importance, (b) goal congruence, (c) possibility, and (d) future expectation for increasing fruit and vegetable intake are positively related to feelings of hope about these dietary improvements. Bivariate correlation analyses revealed that older adults feelings of hope for increasing fruit and vegetable intake were positively correlated with perceptions of importance (r = .62, p<.001), goal congruence (r = .48, p<.001), possibility (r = .48, p<.001), and future expectation (r = .58, p<.001).

Hypothesis three predicted that older adults perceptions of self-efficacy for increasing fruit and vegetable intake is positively related to feelings of hope about these dietary improvements. Bivariate correlation analyses revealed that older adults nutrition self-efficacy was not statistically significantly correlated with feelings of hope for increasing fruit and vegetable intake (r = −0.05, p = 0.73); see Table 2.

A multiple regression analysis was used to predict older adults feelings of hope about increasing fruit and vegetable intake from perceptions of importance, goal congruence, possibility, future expectation, and nutrition self-efficacy. Together, these variables statistically significantly predicted feelings of hope about increasing fruit and vegetable intake, F(5, 52) = 10.88, p<.001, R2 = 0.51. Perceptions of importance, future expectation and nutrition self-efficacy added statistically significantly to the prediction; goal congruence and possibility did not (see Table 3). Of note, the regression coefficient for nutrition self-efficacy was negative. Thus, hypotheses one and three were partially supported.

### 4.2 Hypotheses 2 and 4: Hope for getting more physical activity

Hypothesis two predicted that older adults perceptions of (a) importance, (b) goal congruence, (c) possibility, and (d) future expectation for increasing physical activity are positively related to feelings of hope about physical activity improvements. Bivariate correlation analyses revealed that older adults feelings of hope for getting more physical activity were positively correlated with perceptions of goal congruence (r = .35, p<.01) and possibility (r = 0.34, p < 0.01); feelings of hope for getting more physical activity were not statistically significantly related to perceptions of importance (r = .24, p = 0.07) or future expectation (r = 0.25, p = 0.06).

Hypothesis four predicted that older adults perceptions of self-efficacy is positively related to feelings of hope about physical activity improvements. Bivariate correlation analyses revealed that older adults exercise self-efficacy was not statistically significantly correlated with feelings of hope for getting more physical activity (r = .13, p = .32); see Table 4.

A multiple regression analysis was used to predict older adults feelings of hope about getting more physical activity from perceptions of importance, goal congruence, possibility, future expectation, and exercise self-efficacy. Together, these variables did not statistically significantly predict feelings of hope about getting more physical activity, F(5, 51) = 2.08, p = 0.08, R2 = 0.17; see Table 5. Thus, hypotheses two and four were not supported.

### 4.3 Research question 1

Research question one considered what reasons older adults give for their perceptions of hope related to increasing fruit and vegetable intake and getting more physical activity. The main reasons participants wrote that they were hopeful to make diet or exercise changes were primarily due to their existing knowledge of healthy eating and exercise habits coupled with their desire to lose weight. Participants often indicated knowing what foods were healthy and that they should exercise more; however, they also indicated a need for more support in order to turn these new behaviors into habits that would improve their health outcomes. For example, one 82-year-old woman wrote, “I feel hopeful about improving my diet and exercise routine with your added encouragement and support. I value good nutrition but I can always improve. It is my goal to lose about 25 pounds to return to a normal weight and this program will be a great help in reaching my goal. Thank you so much for your encouragement and good energy.” Additionally, a 62-year-old woman stated, “I’ve tried to increase fruit, [and] vegetables in my diet before but haven’t succeeded as much as desired. I’ve gone through stages, or cycles, where I have done some exercises previously. I haven’t been able to stay consistent.” Likewise, several participants indicated that they are already engaged in exercise but noted that room for improvement. A 62-year-old woman elaborated, “I don’t have a vehicle, so I do walk regularly but it’s so slippery this time of year. I’m trying to increase my indoor exercising [with] YouTube videos. I’m vegetarian and I know how to eat but my motivation tends to not be what it should be.”

Conversely, the top reason participants indicated they lacked hope on this survey question was because of illness or perceived frailty, hindering their ability to engage in physical activity. One 81-year-old woman wrote, “I know I need more physical exercise, [but] I believe I am too physically vulnerable. In my 30 to 50 age range, I was a long-distance runner. I want to be physically stronger, walk, dance, and even run again. My orthopedic doctor said my knees were so terrible, [that] he couldn’t believe I was able to walk around on them. I need both knees replaced.” Additionally, a 72-year-old woman stated, “Recently, I couldn’t stand or take a step due to degeneration of knees, arthritis, allergic reactions, colds, etc. I [now] have to use a scooter for mobility. Having difficulties doing housework and bathing. Have vertigo and fall a lot.” Despite having a desire to make healthful changes, several participants felt hopeless about their ability to increase their physical activity.

Lastly, participants also expressed hope to make these changes due to their desire to enhance their physical well-being. A 77-year-old woman shared her perspective, stating, “I feel participating in this research, I believe I can learn more about eating better and doing more exercise that I can do for my age to live a longer life.” Another woman enumerated her reasons for feeling hopeful, stating “(1) I’m attending this program, feeling more emotionally supported (2) My independent living advocate and I are working on these goals and she’s written an [application] request which has been approved to finance my Tai Chi / Qi Gong classes soon (3) I’m persistent, have a positive vision of my next 30+ years of life.” Some participants noted hesitation and lack of hope, specifically around increasing exercise; however, overall the participants were feeling motivated to make changes and hopeful that a supportive environment would help them do that.

## 5 Discussion

As the proportion of older adults increases in the United States (U.S. Census Bureau 2018) and Alaska (Administration on Aging 2018), it is important to determine how to effectively design messages to promote successful aging via healthy diets and physical activity (Gopinath et al. 2016; Gopinath et al. 2018). This is especially important in Alaska, where older adults face some of the greatest geriatric provider shortages per capita in the country (United Health Foundation 2020; Kaiser Family Foundation 2022) and additional barriers to engaging in these positive health behaviors when compared to those in other areas of the United States (Garrett et al. 2015; Mayeda et al. 2016). Further, persistent disparities in the health of Alaska Native / American Indian peoples and communities underscore the need to promote health and healing (Crouch et al. 2023). Lifespan perspectives suggest that health messages with hope-based linguistic content from PHT (Chadwick 2010; Chadwick 2015) may be particularly beneficial for older adults (Worthington et al., forthcoming; Nussbaum and Worthington 2017). This study therefore examined whether theoretical variables from PHT influence older adults perceptions of hope regarding increasing fruit and vegetable intake and getting more physical activity. The results have important the oretical implications for PHT and practical implications for the specific language features that may be incorporated into messages to lead older adults to feel hopeful and subsequently motivated to engage in healthy changes.

PHT posits that perceptions of importance, possibility, goal congruence, future expectation, and self-efficacy influence feelings of hope (Chadwick 2015). This study found partial support for these theoretical relationships within this sample of older adults in Alaska. Specifically, perceptions of importance and future expectation positively influenced older adults feelings of hope for increasing fruit and vegetable intake. Interestingly, perceptions of goal congruence and possibility did not, and nutrition self-efficacy negatively influenced older adults feelings of hope. The PHT variables related to exercise did not influence older adults feelings of hope for increasing physical activity. There are several potential explanations for these findings.

PHT is a relatively new theory, and significant work is needed to clarify and refine it. It is possible that the influence of the different variables within PHT vary based on the message topic. For example, Chadwick (2015) found that future expectation predicted feelings of hope related to climate change, whereas perceptions of importance, possibility, and goal congruence did not. This study found that several of the PHT variables (importance, future expectation) positively influenced feelings of hope related to fruit and vegetable consumption as expected but not feelings of hope related to increasing physical activity. These differences may be due to older adults interests in the topic itself. For example, past research has found that older adults have low concern for climate change (Pillemer, Nolte, and Cope 2022), whereas older adults have a strong desire for an active, healthy lifestyle (Huijg et al. 2017). Additionally, this study examined the impact of the magnitude of perceptions of importance, possibility, goal congruence, future expectation, and self-efficacy on feelings of hope; however, feelings of hope might actually arise from changes in these perceptions (Chadwick 2015). Feelings of hope might therefore arise if a message leads someone to believe that a future opportunity is more important, possible, and / or goal congruent than they believed previously. Future work that examines whether a change in these perceptions leads to older adults feelings of hope regarding improving their diet and physical activity levels is needed.

This study unexpectedly found that nutrition self-efficacy had a negative influence on perceptions of hope regarding increasing fruit and vegetable intake. One potential rationale for this result may be due to the measure used in this study. The items this study used to assess nutrition self-efficacy were based on the SRAHP (Becker et al. 1993); however, we created our own measure of nutrition self-efficacy using only four items because older adults are at high risk for survey fatigue (Le, Han, and Palamar 2021). The nutrition self-efficacy scale we created in this study assesses overall nutrition self-efficacy with respect to, for example, finding healthy foods, determining a healthy weight, determining which foods are high in fiber content, and so on. The principle of specificity (Ajzen 1988; Ajzen 1991) notes that to best influence an outcome, predictors must relate to a very specific behavior. Thus, to best predict feelings of hope for increasing fruit and vegetable intake, the nutrition self-efficacy items may need to specifically refer to feeling able to find, consume, and prepare fruits and vegetables. Additionally, it may be that the measure we created for this study does not fully capture all relevant aspects of nutrition self-efficacy.

Finally, this data was collected as part of an intervention program, which may have inadvertently suppressed variance in perceptions of importance, possibility, goal congruence, future expectation, self-efficacy, and feelings of hope. Indeed, this data was collected before the participants participated in a 15-week program designed to increase their fruit and vegetable intake and physical activity levels; thus, the high means in these theoretical variables could suggest a ceiling effect based on participants expectations regarding the intervention program and / or a self-selection bias.

Likewise, the written responses to the question about why (or why not) participants felt hopeful about making these changes may further indicate a self-selection bias. Our results and research with other older adults show that they may be more likely to engage in behavior changes when others in their social networks, such as friends and family, are supportive of these changes, especially older women (Litt, Kleppinger, and Judge 2002; Cress et al. 2005; Beverly, Miller, and Wray 2008). Since many of the participants were women who had signed up for a 15-week in-person health education program, their responses to this written question tended to focus on their excitement for the social aspect of the program. Many participants reported feeling hopeful that the supportive environment of the program would increase their motivation for healthy changes. However, not all participants expressed such hope, since some reported that they perceived themselves to be too frail or ill to change their diet or increase activity significantly. This is not surprising, since other research demonstrates that health behavior changes appear more difficult for older adults to maintain, due to a variety of social, emotional, and cognitive determinants (Hill 2006; Schwarzer 2008; Bales and Buhr 2009; Wood and Neal 2016). Older adults may exhibit low intention for diet and exercise changes due to lack of self-efficacy in their ability to make sustained changes (Bardach, Schoenberg, and Howell 2016; Bennett and Gaines 2010; Nilsson, Lundgren, and Liliequist 2012).

The results from this study also have important practical implications. A critical first step in designing hope-based messages for older adults is to determine what factors lead older adults to feel hope. Subsequent steps would then involve determining the specific language features that may be incorporated in messages to lead older adults to feel hopeful about health behavior changes. This study found that perceptions of importance and future expectation led older adults in the sample to feel hope about increasing their fruit and vegetable intake. Therefore, messages designed to increase these older adults feelings of hope to improve their diets should be strategically designed to include specific language features that increases their perceptions that doing so is important and will lead to a better future (see Worthington et al., forthcoming) for specific examples of language features). Including these message features is likely to lead older adults to pay attention to the message, increase their interest in the topic, believe that the message is effective, and intend to perform the recommended behavior (Chadwick 2015).

### 5.1 Limitations

This study has several limitations that must be considered. First, the participants for this study were part of a convenience sample recruited through four older adult housing communities in urban Southcentral Alaska. Alaska is an incredibly diverse state, where the cost of fruits and vegetables is exponentially higher in some areas, and the climate varies substantially across different regions (Bieniek et al. 2012; First Nations Development Institute 2017). This could therefore impact older adults living in different regions to have varying beliefs about the possibility of achieving recommended diet and exercise patterns. Future work is therefore needed to determine if these results generalize to other older adults in urban Alaska. Additionally, these data were cross-sectional and therefore examined associations between the variables of interest; however, this design forestalls the ability to determine if the relationships between PHT predictor variables and feelings of hope are causally related in this study. Future work should therefore examine the relationships between these variables using an experimental or longitudinal design. Finally, there were several limitations with the measures used in this study. The PHT variables were measured using single-item measures, which are preferred over multiple-item measures when survey fatigue is likely (Matthews, Pineault, and Hong 2022). Additionally, nutrition and exercise self-efficacy were measured using scales we created based on the SRAHP. The variables were measured in this way as older adults are at high risk for survey fatigue (Le, Han, and Palamar 2021); however, it is possible that these measures were limited in their ability to fully and accurately capture the study variables.

### 5.2 Conclusions and future directions

Older adults preference for positive language and information underscores the need to determine which factors and subsequent language features elicit positive feelings such as hope. The results from this study suggest that theoretical variables from PHT, specifically importance and future expectation are associated and, therefore, may influence older adults feelings of hope about making healthy dietary improvements. Interventions to increase older adults feelings of hope to make diet changes should therefore consider incorporating language features that elicit these perceptions.

## Figures and Tables

**Table 1:** Participant Demographics (N = 58). Race: Participants were able to select more than one response option; Ethnicity: Several participants chose not to answer these items

	n	%
**Gender**		
Female	49	84.48%
Male	9	15.52%
**Race**		
White / Caucasian	39	67.24%
Alaska Native/American Indian peoples	14	24.14%
Asian	1	1.72%
Black / African American	8	13.79%
Native Hawaiian / Other Pacific Islander	1	1.72%
**Ethnicity**		
Hispanic / Latino(a)	3	5.17%
Not Hispanic / Latino(a)	45	77.59%
**Education Level**		
Some high school (grades 9–12)	4	6.90%
High school graduate or GED	10	17.24%
Vocational or technical school	1	1.72%
Some college	17	29.31%
College graduate	12	20.69%
Post-graduate degree	6	10.34%
Other	8	13.79%
**Income**		
Under $ 25 000	30	51.72%
$ 25 000–$44 999	19	32.76%
$ 45 000–$99 999	3	5.17%
$ 100 000 or above	0	0.00%

**Table 2:** Means (M), standard deviations (SD), and bivariate correlations for variables related to increasing fruit and vegetable intake (*p < 0.05)

Variable	1	2	3	4	5	6	M	SD
1. Importance	-						4.40	0.67
2. Goal Congruence	0.78*	-					4.34	0.64
3. Possibility	0.64*	.59*	-				4.26	0.74
4. Future Expectation	0.65*	0.62*	0.44*	-			4.47	0.66
5. Nutrition Self-Efficacy	0.16	0.20	0.11	0.33*	-		2.95	0.90
6. Hope	0.62*	0.48*	0.48*	0.58*	−.05		4.40	0.75

**Table 3:** Multiple regression analysis to predict older adults feelings of hope about increasing fruit and vegetable intake (*p < 0.05)

Variable	Unstandardized Beta	*β*	p
Importance	0.43	0.39	0.031*
Goal Congruence	−0.14	−0.12	0.464
Possibility	0.15	0.15	0.264
Future Expectation	0.48	0.42	0.003*
Nutrition Self-Efficacy	−0.20	−0.24	0.023*

**Table 4:** Means (M), standard deviations (SD), and bivariate correlations for variables related to to getting more physical activity (*p < 0.05)

Variable	1	2	3	4	5	6	M	SD
1. Importance	-	4.45	0.75					
2. Goal Congruence	0.85*	-	4.41	0.77				
3. Possibility	0.75*	0.69*	-	4.24	0.87			
4. Future Expectation	0.78*	0.80*	0.56*	-	4.59	0.68		
5. Exercise Self-Efficacy	0.41*	0.27*	0.39*	0.29*	-	2.85	0.98	
6. Hope	0.24	0.35*	0.34*	0.25	0.13	-	4.32	0.78

**Table 5:** Multiple regression analysis to predict older adults feelings of hope about getting more physical activity (*p < 0.05)

Variable	Unstandardized Beta	*β*	p
Importance	−0.40	−0.39	0.19
Goal Congruence	0.44	0.44	0.12
Possibility	0.27	0.29	0.14
Future Expectation	0.03	0.03	0.90
Exercise Self-Efficacy	0.04	0.05	0.75

## References

[R1] Administration on Aging. 2018. 2017 profile of older Americans. Report. Washington DC: Administration on Aging, Administration for Community Living, U.S. Department of Health and Human Services. https://acl.gov/sites/default/files/aging%20and%20disability%20in%20america/2017olderamericansprofile.pdf.

[R2] Ajzen Icek. 1988. Attitudes, personality and behaviour. Milton Keynes: Open University Press.

[R3] —. 1991. “The theory of planned behavior.” Organizational Behavior and Human Decision Processes 50 (2): 179–211. 10.1016/0749-5978(91)90020-T.

[R4] Bales Connie W., and Buhr Gwendolen T.. 2009. “Body mass trajectory, energy balance, and weight loss as determinants of health and mortality in older adults.” Obesity Facts 2 (3): 171–178. 10.1159/000221008. 20054222 PMC6516201

[R5] Bardach Shoshana H., Schoenberg Nancy E., and Howell Britteny M.. 2016. “What motivates older adults to improve diet and exercise patterns?” Journal of Community Health 41 (1): 22–29. 10.1007/s10900-015-0058-5. 26159781

[R6] Becker Heather, Stuifbergen Alexa, Oh Hyun Soo, and Hall Sharon. 1993. “Self-rated abilities for health practices: A health self-efficacy measure.” Health Values: The Journal of Health Behavior, Education & Promotion 17 (5): 42–50.

[R7] Bennett Teri, and Gaines Jean. 2010. “Believing what you hear: The impact of aging stereotypes upon the old.” Educational Gerontology 36 (5): 435–445. 10.1080/03601270903212336.

[R8] Russell Bernard, H. 2006. Research methods in anthropology: Qualitative And quantitative approaches. 4th. Lanham: AltaMira Press.

[R9] Bernhold Quinten S. 2021. “The role of media in predicting older adults’ own age-related communication and successful aging.” Mass Communication and Society 24 (1): 1–30. 10.1080/15205436.2020.1743862.

[R10] Beverly Elizabeth A., Miller Carla K., and Wray Linda A.. 2008. “Spousal support and food-related behavior change in middle-aged and older adults living with type 2 diabetes.” Health Education & Behavior 35 (5): 707–720. 10.1177/1090198107299787. 17456857

[R11] Bieniek Peter A., Bhatt Uma S., Thoman Richard L., Angeloff Heather, Partain James, Papineau John, Fritsch Frederick, 2012. “Climate divisions for Alaska based on objective methods.” Journal of Applied Meteorology and Climatology 51 (7): 1276–1289. 10.1175/jamc-d-11-0168.1.

[R12] Buchwald Dedra, Mendoza-Jenkins Veronica, Calvin Croy, McGough Helen, Bezdek Marjorie, and Spicer Paul. 2006. “Attitudes of urban American Indians and Alaska Natives regarding participation in research.” Journal of General Internal Medicine 21 (6): 648–651. 10.1111/j.1525-1497.2006.00449.x. 16808751 PMC1924613

[R13] Burnette Catherine Elizabeth, and Figley Charles R.. 2017. “Historical oppression, resilience, and transcendence: Can a holistic framework help explain violence experienced by Indigenous people?” Social Work 62 (1): 37–44. 10.1093/sw/sww065. 28395035

[R14] Card Noel A. 2015. Applied meta-analysis for social science research. New York: Guilford Press.

[R15] Carstensen LL, Isaacowitz DM, and Charles ST. 1999. “Taking time seriously: A theory of socioemotional selectivity.” American Psychologist 54 (3): 165–181. 10.1037//0003-066x.54.3.165. 10199217

[R16] Chadwick Amy E. 2010. “Persuasive hope theory and hope appeals in messages about climate change mitigation and seasonal influenza prevention.” Doctoral dissertation. https://etda.libraries.psu.edu/catalog/10950.

[R17] —. 2015. “Toward a theory of persuasive hope: Effects of cognitive appraisals, hope appeals, and hope in the context of climate change.” Health Communication 30 (6): 598–611. 10.1080/10410236.2014.916777. 25297455

[R18] Charmaz Kathy. 2006. Constructing grounded theory: A practical guide through qualitative analysis. Thousand Oaks: Sage.

[R19] Cobb Nathaniel, Espey David, and King Jessica. 2014. “Health behaviors and risk factors among American Indians and Alaska Natives, 20002010.” American Journal of Public Health 104 (S3): S481–S489. 10.2105/AJPH.2014.301879. 24754662 PMC4035866

[R20] Cress M. Elaine, Buchner David M., Prohaska Thomas, Rimmer James, Brown Marybeth, Macera Carol, Dipietro Loretta, and Chodzko-Zajko Wojtek. 2005. “Best practices for physical activity programs and behavior counseling in older adult populations.” Journal of Aging and Physical Activity 13 (1): 61–74. 10.1123/japa.13.1.61. 15677836

[R21] Crouch Maria C., Cheromiah Salazar Maredyth B. R., Harris Steven J., and Rosich Rosellen M.. 2023. “Dementia, substance misuse, and social determinants of health: American Indian and Alaska Native peoples’ prevention, service, and care.” Chronic Stress 7:24705470221149479. 10.1177/24705470221149479. PMC9869198 36699807

[R22] Faul Franz, Erdfelder Edgar, Lang Albert-Georg, and Buchner Axel. 2007. “G*Power 3: A flexible statistical power analysis program for the social, behavioral, and biomedical sciences.” Behavior Research Methods 39 (2): 175–191. 10.3758/bf03193146. 17695343

[R23] First Nations Development Institute. 2017. “First Nations Development Institute releases first quarterly results from monitoring food prices, indicating that American Indians and Alaska Natives pay higher costs.” GlobeNewswire, https://www.globenewswire.com/en/news-release/2017/06/05/1008083/0/en/First-Nations-Development-Institute-Releases-First-Quarterly-Results-from-Monitoring-Food-Prices-Indicating-that-American-Indians-and-Alaska-Natives-Pay-Higher-Costs.html.

[R24] Fredrickson BL, and Carstensen LL. 1990. “Choosing social partners: how old age and anticipated endings make people more selective.” Psychology and Aging 5 (3): 335–347. 10.1037//0882-7974.5.3.335. 2242238 PMC3155996

[R25] Fung Helene H., and Carstensen Laura L.. 2006. “Goals change when life’s fragility is primed: Lessons learned from older adults, the September 11 attacks and SARS.” Social Cognition 24 (3): 248–278. 10.1521/soco.2006.24.3.248.

[R26] Garrett Mario D., Baldridge Dave, Benson William, Crowder Jolie, and Aldrich Nancy. 2015. “Mental health disorders among an invisible minority: Depression and dementia among American Indian and Alaska Native Elders.” The Gerontologist 55 (2): 227–236. 10.1093/geront/gnu181. 26035598

[R27] Glaser B, and Strauss A. 1967. Discovery of grounded theory: Strategies for qualitative research. Chicago: Aldine. 10.4324/9780203793206.

[R28] Gopinath Bamini, Kifley Annette, Flood Victoria M., and Mitchell Paul. 2018. “Physical activity as a determinant of successful aging over ten years.” Scientific Reports 8 (1): 10522. 10.1038/s41598-018-28526-3. 30002462 PMC6043510

[R29] Gopinath Bamini, Russell Joanna, Kifley Annette, Flood Victoria M., and Mitchell Paul. 2016. “Adherence to dietary guidelines and successful aging Over 10 years.” The Journals of Gerontology: Series A 71 (3): 349–355. 10.1093/gerona/glv189. 26503376

[R30] Hernandez Silvia C., and Overholser James C.. 2021. “A systematic review of interventions for hope/hopelessness in older adults.” Clinical Gerontologist 44 (2): 97–111. 10.1080/07317115.2019.1711281. 31913808

[R31] Hill James O. 2006. “Understanding and addressing the epidemic of obesity: An energy balance perspective.” Endocrine Reviews 27 (7): 750–761. 10.1210/er.2006-0032. 17122359

[R32] Howell Britteny M., Seater Mariah, Davis Kathryn, and McLinden Daniel. 2022. “Determining the importance and feasibility of various aspects of healthy ageing among older adults using concept mapping.” Ageing & Society 42 (6): 1403–1421. 10.1017/S0144686X20001580.

[R33] Huijg Johanna M., van Delden AEQ, van der Ouderaa Frans J. G., Westendorp Rudi G. J., Slaets Joris P. J., and Lindenberg Jolanda. 2017. “Being active, engaged, and healthy: Older persons’ plans and wishes to age successfully.” The Journals of Gerontology: Series B 72 (2): 228–236. 10.1093/geronb/gbw107. 27591730

[R34] IBM Corporation. 2020. SPSS. Computer program. V. 27. Aramonk.

[R35] Kaiser Family Foundation. 2022. Primary care health professional shortage areas (HPSAs). Report. San Francisco: Kaiser Family Foundation. https://www.kff.org/other/state-indicator/primary-care-health-professional-shortage-areas-hpsas/.

[R36] Lang Frieder R., and Carstensen Laura L.. 2002. “Time counts: Future time perspective, goals, and social relationships.” Psychology and Aging 17 (1): 125–139. 10.1037/0882-7974.17.1.125. 11931281

[R37] Le Austin, Han Benjamin H., and Palamar Joseph J.. 2021. “When national drug surveys “take too long”: An examination of who is at risk for survey fatigue.” Drug and Alcohol Dependence 225:108769. 10.1016/j.drugalcdep.2021.108769. 34049103 PMC8282613

[R38] Litt Mark D., Kleppinger Alison, and Judge James O.. 2002. “Initiation and maintenance of exercise behavior in older women: Predictors from the social learning model.” Journal of Behavioral Medicine 25 (1): 83–97. 10.1023/a:1013593819121. 11845560

[R39] Liu Xiaomei, Shuster Michael M., Mikels Joseph A., and Stine-Morrow Elizabeth A. L.. 2019. “Doing what makes you happy: Health message framing for younger and older adults.” Experimental Aging Research 45 (4): 293–305. 10.1080/0361073X.2019.1627491. 31188722

[R40] Matthews Russell A., Pineault Laura, and Hong Yeong-Hyun. 2022. “Normalizing the use of single-item measures: Validation of the single-item compendium for organizational psychology.” Journal of Business and Psychology 37 (4): 639–673. 10.1007/s10869-022-09813-3.

[R41] Mayeda Elizabeth Rose, Glymour M. Maria, Quesenberry Charles P., and Whitmer Rachel A.. 2016. “Inequalities in dementia incidence between six racial and ethnic groups over 14 years.” Alzheimer’s & Dementia 12 (3): 216–224. 10.1016/j.jalz.2015.12.007. PMC4969071 26874595

[R42] Morse Janice M. 2015. “Critical analysis of strategies for determining rigor in qualitative inquiry.” Qualitative Health Research 25 (9): 1212–1222. 10.1177/1049732315588501. 26184336

[R43] Neuendorf Kimberly A. 2002. The content analysis guidebook. Thousand Oaks: Sage.

[R44] Nicklett Emily J., and Kadell Andria R.. 2013. “Fruit and vegetable intake among older adults: A scoping review.” Maturitas 75 (4): 305–312. 10.1016/j.maturitas.2013.05.005. 23769545 PMC3713183

[R45] Nilsson Ingeborg, Lundgren Anna Sofia, and Liliequist Marianne. 2012. “Occupational well-being among the very old.” Journal of Occupational Science 19 (2): 115–126. 10.1080/14427591.2011.595894.

[R46] Notthoff Nanna, and Carstensen Laura L.. 2014. “Positive messaging promotes walking in older adults.” Psychology and Aging 29 (2): 329–341. 10.1037/a0036748. 24956001 PMC4069032

[R47] Notthoff Nanna, Klomp Peter, Doerwald Friederike, and Scheibe Susanne. 2016. “Positive messages enhance older adults’ motivation and recognition memory for physical activity programmes.” European Journal of Ageing 13 (3): 251–257. 10.1007/s10433-016-0368-1. 28804382 PMC5550637

[R48] Nussbaum Jon F., and Worthington Amber K.. 2017. “Lifespan and developmental considerations in health and risk message design.” In Oxford Research Encyclopedia of Communication. Oxford: Oxford University Press. 10.1093/acrefore/9780190228613.013.318.

[R49] Pillemer Karl A., Nolte Julia, and Cope Marie Tillema. 2022. “Promoting climate change activism among older people.” Generations: Journal of the American Society on Aging 46 (2): 1–16. https://www.jstor.org/stable/48697100.

[R50] Pruchno Rachel. 2015. “Successful aging: Contentious past, productive future.” Gerontologist 55 (1): 1–4. 10.1093/geront/gnv002. 26035905 PMC4986598

[R51] QSR International. 2018. NVivo. Computer program. V. 12 Pro. Burlington, MA.

[R52] Reed Andrew, and Carstensen Laura. 2012. “The theory behind the age-related positivity effect.” Frontiers in Psychology 3. 10.3389/fpsyg.2012.00339. PMC3459016 23060825

[R53] Reed Andrew, Chan Larry, and Mikels Joseph A.. 2014. “Meta-analysis of the age-related positivity effect: Age differences in preferences for positive over negative information.” Psychology and Aging 29 (1): 1–15. 10.1037/a0035194. 24660792

[R54] Schwarzer Ralf. 2008. “Modeling health behavior change: How to predict and modify the adoption and maintenance of health behaviors.” Applied Psychology 57 (1): 1–29. 10.1111/j.1464-0597.2007.00325.x.

[R55] Shamaskin Andrea M., Mikels Joseph A., and Reed Andrew E.. 2010. “Getting the message across: Age differences in the positive and negative framing of health care messages.” Psychology and Aging 25 (3): 746–751. 10.1037/a0018431. 20677886

[R56] Sparling Phillip B., Howard Bethany J., Dunstan David W., and Owen Neville. 2015. “Recommendations for physical activity in older adults.” BMJ: British Medical Journal 350:h100. 10.1136/bmj.h100. 25608694

[R57] U.S. Census Bureau. 2018. The U.S. joins other countries with large aging populations. Report. Washington DC: U.S. Census Bureau. https://www.census.gov/library/stories/2018/03/graying-america.html.

[R58] United Health Foundation. 2020. 2019 senior report: Alaska. Report. Minnetonka: United Health Foundation. https://www.americashealthrankings.org/learn/reports/2019-senior-report/state-summaries-alaska.

[R59] Vincent GK, and Velkoff VA. 2010. The next four decades: The older population in the United States: 2010 to 2050. Report P25–1138. Washington DC: U.S. Census Bureau. https://www.aarp.org/content/dam/aarp/livable-communities/old-learn/demographics/the-next-four-decades-the-older-population-in-the-united-states-2010-2050-aarp.pdf.

[R60] Watson Kathleen B., Carlson Susan A., Gunn Janelle P., Galuska Deborah A., Ann O’Connor Kurt J. Greenlund, and Fulton Janet E.. 2016. “Physical inactivity among adults aged 50 years and older — United States, 2014.” Morbidity and Mortality Weekly Report 65 (36): 954–958. 10.15585/mmwr.mm6536a3. 27632143

[R61] Wood Wendy, and Neal David T.. 2016. “Healthy through habit: Interventions for initiating & maintaining health behavior change.” Behavioral Science & Policy 2 (1): 71–83. 10.1177/237946151600200109.

[R62] Worthington Amber K., Howell Britteny M., Golden Dale M., and Mahanna Allexis. Forthcoming. “Creating a student-led healthy aging program for diverse older adults in Alaska using Persuasive Hope Theory.” In Health communication, language, and social action across the lifespan, edited by Fisher Carla, Fowler Craig, Krieger Janice, Pitts Margaret, Worthington Amber K., and Nussbaum Jon. New York: Peter Lang.

